# Gaze Zone Classification for Driving Studies Using YOLOv8 Image Classification

**DOI:** 10.3390/s24227254

**Published:** 2024-11-13

**Authors:** Frouke Hermens, Wim Anker, Charmaine Noten

**Affiliations:** Department of Computer Science, Open University of the Netherlands, 6419 AT Heerlen, The Netherlands; wimanker@outlook.com (W.A.); charmaine211@hotmail.com (C.N.)

**Keywords:** gaze zone, drivers, YOLOv8, image classification

## Abstract

Gaze zone detection involves estimating where drivers look in terms of broad categories (e.g., left mirror, speedometer, rear mirror). We here specifically focus on the automatic annotation of gaze zones in the context of road safety research, where the system can be tuned to specific drivers and driving conditions, so that an easy to use but accurate system may be obtained. We show with an existing dataset of eye region crops (nine gaze zones) and two newly collected datasets (12 and 10 gaze zones) that image classification with YOLOv8, which has a simple command line interface, achieves near-perfect accuracy without any pre-processing of the images, as long as a model is trained on the driver and conditions for which annotation is required (such as whether the drivers wear glasses or sunglasses). We also present two apps to collect the training images and to train and apply the YOLOv8 models. Future research will need to explore how well the method extends to real driving conditions, which may be more variable and more difficult to annotate for ground truth labels.

## 1. Introduction

Distraction is a major risk factor for traffic accidents [1]. Several studies found that activities like using a phone or looking at objects at the roadside substantially increase the risk of an accident or near-accident, particularly if drivers look away from the road [2,3,4]. In an attempt to reduce traffic accidents due to distracted driving, research on Advanced driver-assistance systems (ADAS) has therefore (among other aspects) focused on identifying where drivers look [5,6].

Identifying the gaze direction in drivers can be performed in two broad ways: (1) identifying the exact direction in which the driver is looking (i.e., estimating the gaze vector) or (2) identifying which region a driver is looking at (e.g., determining whether the driver is looking straight ahead, in the rear mirror, or over the shoulder). The first task is much more challenging to perform and often requires dedicated eye tracking technology, but also gives more detailed information. Such detailed information may, however, not always be necessary. Eye tracking studies typically perform a regions of interest analysis [7,8], in which the continuous gaze position is converted to discrete regions of interest. If determining the region of interest gazed at by drivers is the aim of the study (e.g., [9,10]), one can instead directly try to measure which gaze zones drivers look at without the intermediate step of determining the gaze vector.

Broadly two general groups of users for such gaze zone detection systems can be identified: (1) developers of ADAS technology who want to be able to give a warning to a driver during distraction [11,12] and (2) road safety researchers, who would like to study where drivers look when they are driving, for example, while changing lanes [13,14,15].

The goals of these two groups in terms of automatic gaze zone detection are not the same. The ADAS developers desire a method that works well for all drivers and under most driving conditions (universal gaze zone classification [16]). This is a challenging task because of the large diversity in the visual appearance of drivers, and how they shift attention (e.g., head turning or eye movements [17]). The system must also work under a broad range of situations (rain, sun, evening, morning [18]). ADAS developers typically have technically skilled people available to develop complex computer algorithms for this task. They are less likely to share the code because of commercial interest and competition in the field.

In contrast, road safety researchers can often focus on particular drivers and particular driving conditions. For example, the UDRIVE project [19,20,21,22] collected data from 290 drivers across seven countries, which included 200 car drivers in four countries and 50 truck drivers in one country [20]. Each vehicle was fitted with five to eight cameras and data were collected across 21 months of driving. Another naturalistic driving project collected 43,000 h of driving data across 100 drivers across a 12-month period [3]. Initially, manual annotation of the gaze direction of the 290 UDRIVE drivers was planned [20], but it is unclear whether this annotation was carried out. Gaze zone annotation has been performed for the 100 drivers dataset [3,23].

Other studies used smaller datasets to keep the annotation work within bounds. For example, Peng and colleagues [24] annotated on-road and off-road attention for 5 s or 8 s clips of 24 drivers, resulting in a total of 71 clips for analysis. Tivesten et al. [25] manually coded around 1.5 h of driving for on-road and off-road gaze behaviour. Seppelt et al. [23] made use of the 100 drivers dataset [3] from which 828 sections each of 40 s were manually coded for 13 gaze zones and two categories of missing data (eyes closed and no video). In addition to being extremely labour intensive, a recent study examining the accuracy of such manual annotation showed that human annotators struggle to annotate the correct direction [26]. More worryingly, this study also found that annotators could be in agreement, but collectively wrong in their annotations.

Road safety researchers would therefore benefit from automatic gaze annotation for two reasons: (1) it would make annotation of naturalistic driving data much more feasible and (2) it might circumvent the problem that human annotators struggle to provide accurate annotations [26]. In contrast to ADAS developers, road safety researchers can annotate the images offline (after data collection) and annotation does not need to be performed at a high speed (it can be slower than the frame rate of the recorded video). Because they recruit their research participants, they can also ask these participants for additional recordings in which participants look at a range of instructed gaze directions with the aim of building a personalised gaze zone classifier [16]. As road-safety research groups do not always have highly technically skilled people on their teams, ideally, the method should be relatively easy to use.

We here propose a method that we think can be used by road safety researchers. We suspect that it is (1) easy to use and (2) we demonstrate that it can achieve an extremely high annotation accuracy, even for 10 gaze zones or more (conditions under which human annotators struggle [26]). The method is inspired by the idea of a calibration stage used by eye trackers [27,28]. While there are methods for calibration-free eye tracking [29,30], often tracking is improved by asking a participant to look at a series of targets before starting to collect the eye tracking data. Recordings of the participants’ eyes and face for these targets serve as anchor points to estimate where participants are looking during the remainder of the study.

In our proposed method, we ask drivers to look repeatedly towards different gaze zones (e.g., ‘rear mirror’, ‘over the left shoulder’) and record images during these intervals. The idea of training a model for a specific combination of a driver and driving conditions is not entirely new. It was previously proposed to be able to perform gaze zone detection on a mobile phone [31] (typically, less computing power is available on phones compared with computers), but then combined with the estimation of head pose, which may be challenging for road safety researchers. We here instead propose to use image classification with the recently introduced YOLOv8 package from Ultralytics [32]. Our main reason for using this package is that it is easy to use: training a model involves four lines of Python code and can even be performed from the command line. If the method can be shown to achieve high accuracy, this will allow the method to be used by more researchers, some of whom may not be experts in the field of computer vision [33]. At the time of writing, YOLOv8 was the latest version that was offering image classification, which was the reason for focusing on YOLOv8 rather than newer YOLO versions [34] (and, to anticipate the results, we found that YOLOv8 achieves a very high performance).

Using the proposed method, we test a set of research questions: (1) What accuracy does YOLOv8 image classification achieve when trained and tested on the same driver? (2) What accuracy is achieved when a model is trained on one driver and then applied to images of another driver? (3) What accuracy is obtained when a model is trained on more drivers and then applied on images of those drivers? (4) What accuracy does YOLOv8 achieve on images of drivers wearing glasses or sunglasses? (5) Can the proposed method also be used in other contexts, such as measuring visual and social attention [35]? To address these questions, we make use of an existing dataset [36] and images that we collected using our own newly developed app (which we make available). Across these different datasets, we consistently find that near-perfect gaze zone detection can be obtained with YOLOv8, as long as images that resemble the target conditions and driver are included during training.

## 2. Related Work

In the following, we discuss methods for annotation (needed to create a dataset for training computer vision models), existing datasets, and existing approaches to gaze zone classification. For applications in road safety research, we would like to refer to the excellent overview by Sharma et al. [37].

### 2.1. Annotation Methods

Broadly there are three annotation methods to label the driver images. In the first method drivers are instructed to look at target regions under controlled conditions, for example in a stationary car or in a driving simulator [36,38,39,40,41,42]. This method has the advantage that labelling is automatic: each video frame is associated with a particular instruction (assuming that drivers follow the instructions) and can be automatically labelled for that gaze zone. The disadvantage of this method is that recordings may be different from naturalistic driving conditions. Instructed looking might differ from how drivers look around in naturalistic driving, and a stationary car or driving simulator (a safe environment) may lead to different gaze behaviour than under normal, more risky, driving conditions.

The second approach, which is performed during naturalistic driving, asks drivers to glance in a particular direction [18,43] and to confirm looking in that direction by naming the gaze zone [43] or to point a head-mounted laser pointer to a target inside the cabin [41]. This approach has the advantage that more realistic driving conditions are obtained. Looking while driving, however, is risky and drivers may therefore only briefly glance in the various directions. It is not entirely clear how naturalistic such glance behaviour is.

Third, drivers are recorded during naturalistic driving and human annotators label the video frames for gaze direction [44,45,46,47,48,49,50]. The advantage of this approach is that natural gaze behaviour is recorded. A disadvantage is that annotation is labour intensive, and that human annotation is unlikely to be extremely accurate and may be subject to biases [26].

One paper appears to have used a combination of methods [51], where sensors attached to the head were used to estimate the head’s 3D orientation. These data during naturalistic driving were then clustered into the number of gaze zones and each cluster was then assigned a label by comparing the head’s 3D orientation during instructed gazing in those directions. While this method may provide the best of all worlds, it may be less feasible for road safety due to the technical requirements.

### 2.2. Datasets

The published literature has several gaze zone driver datasets [12,38,43,44,49,52,53,54,55,56]. These datasets, however, are often difficult to acquire [47]. We could download six of the datasets without the need to fill out a form and send it to the authors. These were the Lisa2 dataset [36], the SynDD1 dataset [38], which were both available through the Kaggle platform, DrivFace [57], available through the UCI machine learning repository, 55 rides (with data stored as Matlab files, meaning that you need to convert the files first if you do not have Matlab, [46]), Brain4cars [54], which also uses Matlab files, and the ‘look both ways’ dataset [56], in which drivers wear mobile eye tracking glasses and the data indicate gaze coordinates, not gaze zones. Sometimes the open-access repositories seemed to have missing data, such as for the Lisa2 data set on Kaggle (with fewer drivers than described in the paper) or the SynDD1 dataset (where annotations seemed to be missing for several of the drivers). Some of the datasets seem to have been commercialised and no longer available. This makes benchmarking the various models and approaches difficult, which often leads to authors collecting their own datasets [47].

### 2.3. Previous Modelling Approaches

Past approaches to predict gaze zone labels on the basis of driver images can be divided into two broad categories [37,47]. The first type of approach [16,17,31,42,48,50,58,59,60,61,62] involves the extraction of features from the image (e.g., facial landmarks, the edges of the face, estimates of the head pose—Euler angles), which are then used to predict the gaze direction of the driver, often with traditional machine learning models [17,18,31,39,48,51]. This approach is sometimes called the conventional appearance-based method [37]. Generally, it is found that when both features of the head and features of the eyes are entered into the model, performance improves [17,60]. The advantage of such feature-based methods is that the models are often more robust against variations in the recordings (e.g., lighting conditions, the seat height, the position of the camera) and variations in the drivers (their height or the way that they shift their gaze [17]). This comes at a cost that models are often more difficult to construct and implement.

The second type of approach directly maps the images onto the labels [18,44,49,63,64,65], known as end-to-end [47] or an appearance-based method with deep learning [37]. The specific computer vision task involved is called image classification: the algorithm learns which label to assign to what kinds of images. While this may seem to be a black box method, there are techniques available to reveal what regions of images are used to perform the task [66,67]. End-to-end methods can be applied to the entire image, but often regions are first extracted, such as the face or eyes region, before they are processed. For example, Naqvi et al. [63] extracted the regions for the left eye, the right eye, the face, the pupil of the left eye, and the pupil of the right eye in near-infrared images of the driver and entered these into a deep neural network (a convolutional neural network or CNN) for processing. Likewise, Rangesh and colleagues [68] used an eye region crop and then applied the CycleGAN method to remove the glasses from the image before using image classification on the resulting images. The advantage of end-to-end models is that they are relatively easy to implement, particularly when the entire image is used as input, but this comes at the downside of being more susceptible to variations in the driving conditions and driver features.

Sharma and colleagues [37] distinguish a third separate category, in which a 3D geometric model of the eyes is constructed. This method is often used in combination with a camera close to the face or eyes, but can also be used in a remote setup [16,62,69]. The method can be highly accurate, but mostly under controlled conditions. It is also more complicated to implement. Because the shape of the eye differs between observers, the method does not easily generalise to new drivers. Finally, the method suffers from moments when the eye is occluded (for example, during blinks), because there is no signal to analyse during those moments [37].

The overview by Camberg and colleagues [47] shows that the performance of existing models ranged from around 80% to around 97% correct (Table I in [47]). The best performing previous study in their overview [64] used the end-to-end approach, but studies were difficult to compare because many used different datasets, with different numbers of gaze zones, collected under different driving conditions, and using different methods for model evaluation (leave-one-out or splitting between training and evaluation sets). Leave-one-out is better suited for feature-based models because training of these models is relatively computationally inexpensive compared with end-to-end models (the leave-one-out method requires repeated training of the model). For a better comparison, Camberg et al. [47] therefore constructed a new dataset and tested various approaches on this dataset so that performance could be directly compared. The results indicate that, when there are limited training data, the feature-based approach may provide results that better generalise across drivers (i.e., the method is used to predict gaze zones for yet unseen drivers). Feature-based methods were also found to be less susceptible to differences in the camera position than end-to-end methods [47].

## 3. Methods

### 3.1. Datasets

We made use of three datasets. The first dataset was downloaded from the Kaggle data science platform and consists of a part of the Lisa2 dataset [68]. The reason for using the dataset from Kaggle was that the links to the original location on Google Drive (links were found on this Github page: https://github.com/arangesh/GPCycleGAN, accessed on 10 November 2024) no longer worked. The other two (smaller) datasets we created ourselves by recording ourselves and a third person using an app that we developed for our present research and make available for other researchers to use (more details later).

**Figure 1:**
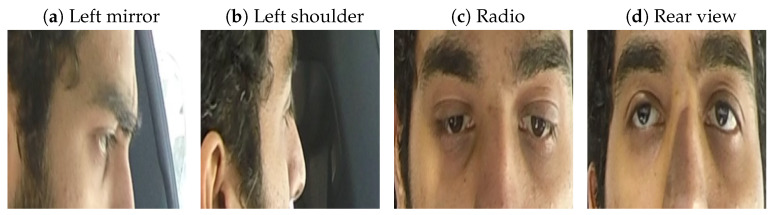
Four images from one of the five drivers in the Lisa2 dataset [36].

The Lisa2 dataset on Kaggle (https://www.kaggle.com/datasets/yousefradwanlmao/driver-gaze-rgb-dataset-lisa-v1-v2, accessed on 10 November 2024) contains images of five (of the original 13 [36]) drivers who were asked to look into nine different directions. Figure 1 shows examples of images of one of the drivers, where it can be seen that the images provide a close crop of the eye region of the driver. Because the dataset contains images of the drivers with and without glasses, we assume that these images were from the study [36] in which participants were instructed to gaze at the different gaze zones during daytime and nighttime conditions, rather than during naturalistic driving. The dataset on Kaggle seems to contain only daytime images, which may have been collected with an infra-red camera [36].

We found that in the dataset the number of images varied slightly per combination of driver, gaze zone, and glasses condition. On average there were 338 images per combination. We split these into on average 279 training images (82.5%) and 59 validation images (17.5%) by moving all the filenames containing the digit zero from the training set to the validation set (thereby avoiding temporally adjacent images in the validation set). We entered the images into the YOLOv8 image classification model without any further pre-processing or data augmentation. Past studies used GANs to artificially remove the glasses [36,68,70], but this is a fairly complex operation and we wanted to determine what accuracy YOLOv8 image classification could achieve without such pre-processing. Because we trained a large number of models and training of each model took around 5 to 7 min each on the hardware that we had available (Google Colab and an GeForce RTX 750 Ti card), we used a single split between training, validation, and test. The convergence of results across the various datasets suggests that similar results would have been obtained for different splits between training and test.

The Lisa2 dataset from Kaggle had a few possible limitations. First, it had crops of the eye region. This removes possible information of the head and body direction of the drivers. Second, there were relatively few images from each driver (an average of 338 per driver and gaze zone), which made us decide to only create a training and a validation set for this dataset, and not a separate test set (of unseen images). Third, the glasses often blocked the view of the pupil region of the eye, but not always, and therefore the comparison between glasses and no glasses does not fully distinguish between detection with and without eye-gaze direction.

**Figure 2:**
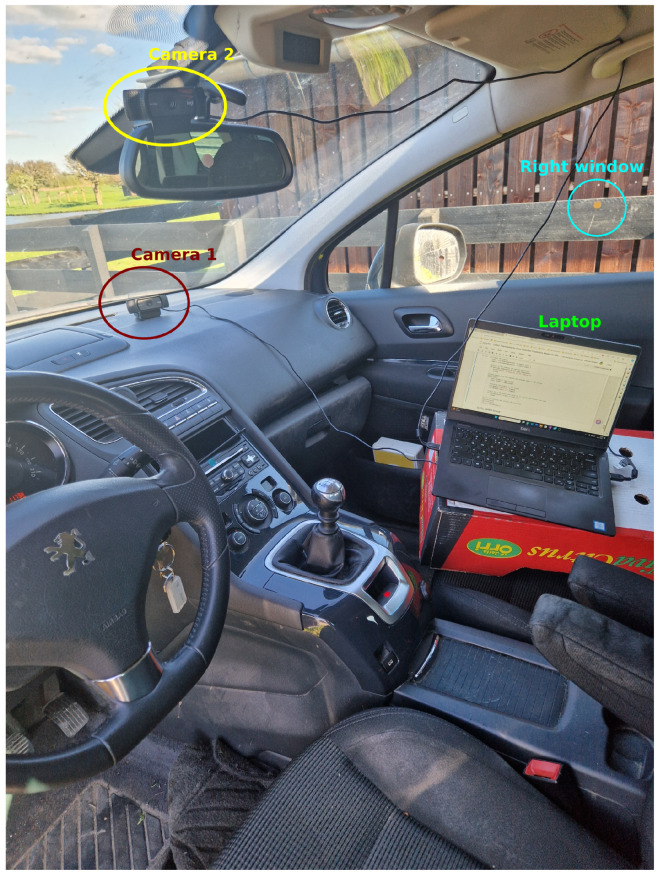
Photograph of the setup. Two webcams were attached to a laptop controlling data collection and placed on the driver seat. Little round stickers in different colours served to help the participant to fixate on different gaze zones. The position of the sticker for the right window is indicated. Other stickers inside this image are for the speedometer, the centre console, and the right mirror.

To address these possible issues, we therefore created our own datasets, for which we developed an app that we also share so the other researchers can use it to collect their own datasets (more information later). The first of these datasets was collected inside a stationary car (see Figure 2), where we instructed the two participating drivers (one male and one female, both in their early 50 years and with more than 30 years of driving experience) to look into one of twelve gaze zones, based on Dutch guidelines for driving instructions (see Table 1).

The images were collected inside a Peugeot 5008 parked in a driveway (Figure 2). Two Logitech C920 webcams (Logitech Europe S.A., Lausanne, Switzerland) were used, both connected to a laptop placed on the passenger seat (one camera above the rearview mirror and another on the dashboard in front of the passenger seat, see Figure 2). The direction of both cameras was adjusted to capture the head and upper body of the driver across the different gaze directions. Small circular stickers in different colours were used to indicate the different target gaze locations (see Table 1). Each of the twelve instructions were presented 10 times (a computer voice read out the instructions entered into the apps, so no other person was needed for these instructions), in a random order, while both cameras recorded the driver for 5 s for each instruction, and stored the videos at a resolution of 640 by 480 pixels at 30 frames per second in the AVI format.

The male driver was recorded across two sessions: once with and once without sunglasses. The female driver was recorded once, without sunglasses. We created additional versions of these datasets of half the image size by reducing the images from 640 by 480 to 320 by 240 pixels.

For training of the YOLOv8 models, we extracted frames from the recorded videos at equal intervals such, that per gaze zone, 608 training, 202 validation, and 202 test images were available. Frames were extracted in equal numbers from both webcams, and from each of the 10 repetitions of each gaze zone, thereby creating variation in the angle of the webcam and the way the driver looked at the target.

**Figure 3:**
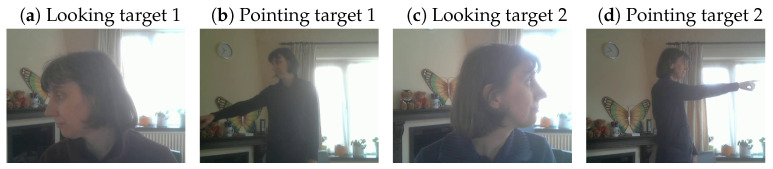
Examples of images of looking and pointing in a different context. A total of 10 different targets were selected around the screen that the webcam was attached to and other parts of the room. Note that in between recording sessions the actor changed the blue jacket for a red jacket.

A similar dataset was created inside the living room of one of the authors, where it was investigated whether the same methodology could be used to classify gaze zones in a different context and for pointing gestures (Figure 3 and Table 2). For this dataset, ten different objects were used that were positioned around the computer screen, to which one webcam (Logitech HD 720p, Logitech Europe S.A., Lausanne, Switzerland) was attached. Some of these objects were more remote from the screen (door handle, door stop, plant) and some close to the screen and each other (webcam, phone, notebook, computer, picture, socket, and cup).

Because the male driver in the in-car dataset was wearing different jackets while recording the images with and without sunglasses, we also used the living room recordings to determine the effects of wearing a different jacket. We therefore recorded images of the actor in the living room with both a blue and a red jacket (see Figure 3 for examples). This allowed us to investigate whether a model trained on a blue jacket images performed well on red jacket images, and vice versa, and whether a model trained on both types of jackets performed well on both types of images. For each gaze zone we extracted 138 train images, 45 validation images, and 45 test images.

### 3.2. Model Training

Because of its ease of use, we used image classification from YOLOv8 by Ultralytics [32]. The YOLOv8 image classification neural network structure can be divided into a backbone, neck, and head [71]. The backbone uses a pre-trained neural network to extract features from the image. The neck merges these features using structures called feature pyramid networks (FPNs). These combined features are then fed through the head, which assigns feature class labels alongside with their probabilities. Important advantages of YOLOv8 are its ease of use, often excellent performance, and fast inference time [33]. Like other deep learning methods, a graphical processing unit (GPU) is needed to train a model.

We entered the original images into the model without any form of pre-processing. During training, the weights of the smallest (’nano’) model that was originally pre-trained on the ImageNet dataset [72] were adjusted (a process known as fine-tuning). For all three datasets, we used the default set of hyperparameters of YOLOv8, except for the number of epochs. We found that all models achieved their best performance well within 10 epochs, and we therefore trained each model for 10 epochs only. An important advantage of YOLOv8 image classification is that, while a graphics card is highly recommended for training the models, this does not need to be a high-end graphics card. The model for images 640 by 480 pixels in size required 4Gb of GPU RAM, whereas the model for images 320 by 240 pixels could be trained with 2Gb of GPU RAM.

## 4. Results

### 4.1. Lisa2

**Figure 4:**
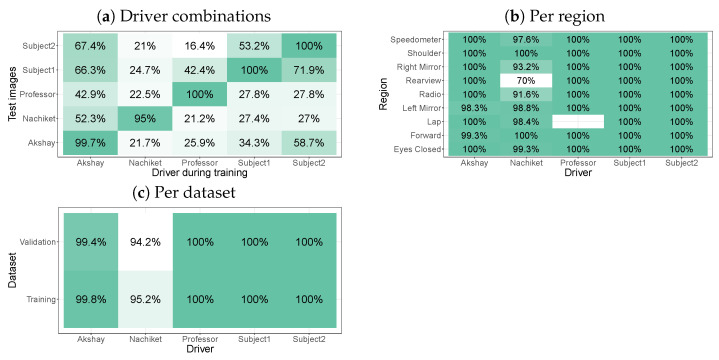
Accuracy per model trained on individual drivers for the Lisa2 dataset without glasses. Accuracy is defined as the percentage of predictions that agree with the annotated label (also known as the ’top1’ accuracy).

In our first test with the Lisa2 dataset, we trained individual models for each driver without glasses and applied these models to all of the images of the same driver or to all of the images of other drivers. Figure 4a shows that a near-perfect classification performance (i.e., the label with the highest probability, the predicted label, is almost always the annotated label) was obtained when the driver during training and inference was the same. In contrast, models trained on one driver did not generalise well to images of another driver.

Figure 4b plots a portion of these data (the data for which the driver agreed between training and validation), but split per region. This graph reveals that, for one of the drivers, there were no images for the ’lap’ label (in the original paper, ’lap’ and ’eyes closed’ were taken together [49]). For one of the drivers (’Nachiket’), the model had a lower performance (70% correct) for the rear view.

The accuracy thus far combined the training and validation images. Figure 4c shows that the performance was similar across these two image sets. A somewhat lower performance was observed for one of the drivers (’Nachiket’) on both image sets, but a near-perfect performance was found for the other drivers.

**Figure 5:**
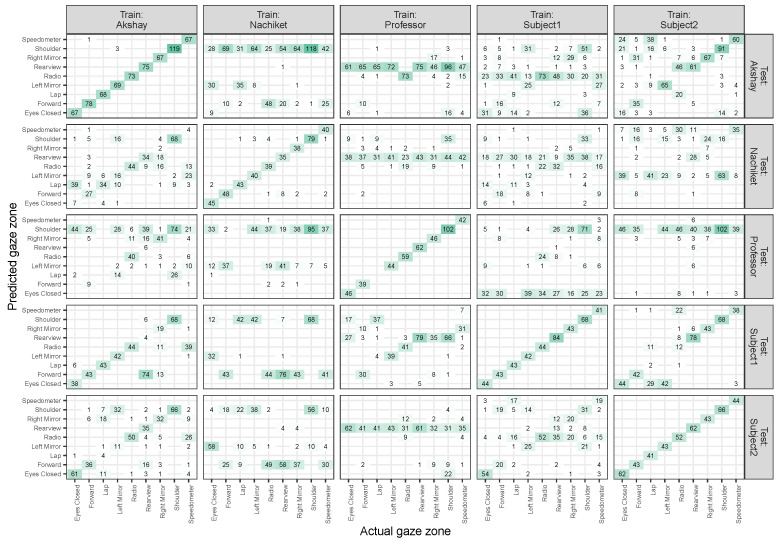
Confusion matrices for each combination of the driver during training and the driver used for the test images, based on the validation sets.

Zones and drivers in the dataset differed in their numbers of images and it is therefore also important to explore the confusion matrix. Defining other metrics, such as area under the ROC curve, area under the precision–recall curve, precision, and recall, is more complicated due to the multi-class nature of the classification problem, and we therefore instead present counts on the validation set in Figure 5. This shows that, when there was an overlap between drivers between training and test, almost all counts were on the diagonal (in agreement with the high accuracy numbers). When there was a mismatch between drivers between training and test, class confusions took place between many of the categories. There were zones that were predicted more often, such as the shoulder, rearview, and forward.

One strategy to gain better performance on multiple drivers from a single model is to train a model on images from multiple drivers. We therefore increased the number of drivers in the training set from one to two, to three, to four, and to all five drivers. We entered the drivers one by one into the model in the following order: Akshay, Nachiket, Professor, Subject1, Subject2. This means that the model trained with three drivers, for example, was trained on images from Akshay, Nachiket, and Professor.

**Figure 6:**
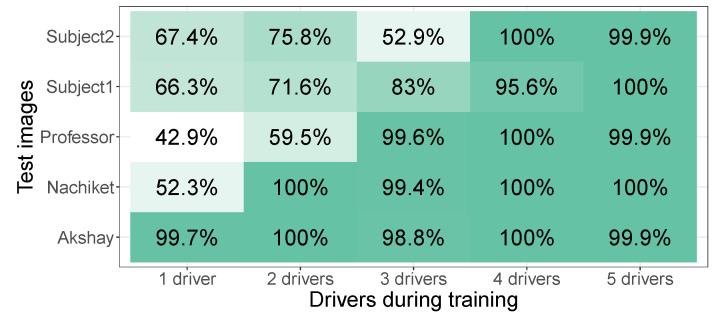
Accuracy per driver on models trained on different numbers of drivers for the Lisa2 dataset without glasses.

Figure 6 shows that high classification accuracy was obtained as long as the model had seen a driver during training. For example, driver Nachiket was added to the model for two drivers, while this driver was not seen during the training of the model on one driver. Adding images of Nachiket during training led to a jump in the accuracy from 52.3% to 100% for his images.

Interestingly, the overall performance hardly decreased by including more drivers (but at the cost of longer training times). This means that, in addition to using one model per driver, a second strategy is to train a model for all drivers that need to be annotated. Note that the data also suggest that models that are trained on more drivers tend to do better on drivers that are not included in the training. For example, the model with four drivers achieves 100% for Subject2, who was not involved during training. The model with two drivers only achieves 75.8% for this same driver.

**Figure 7:**
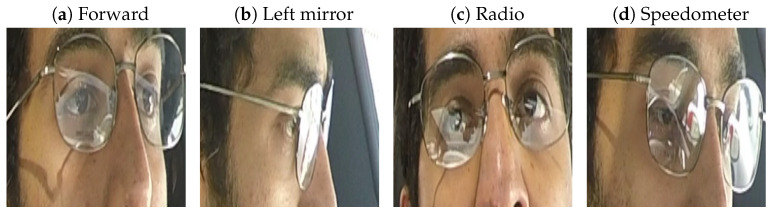
Four images from one of the five drivers in the Lisa2 dataset, now with glasses.

**Figure 8:**
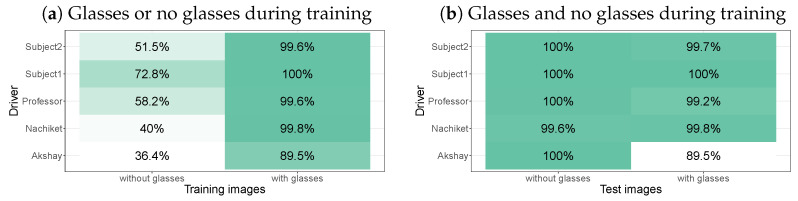
(**a**) Accuracy per driver on images with glasses when trained on images without glasses or images with glasses. (**b**) Accuracy per driver on images with and without glasses when trained on images with and without glasses. Images are from the Lisa2 dataset.

The Lisa2 dataset on Kaggle also contains images of the drivers with glasses (see Figure 7 for examples). Figure 8a shows that the original models, trained on each of the drivers without glasses, perform poorly on the images with glasses, even when they involve the same driver. When trained on the images with glasses, performance improves significantly (comparison of the fits of mixed effects models: $\chi^{2}\left(1\right)$ = 13.6, *p* = 0.00022). Only for Akshay does the performance stay relatively low at 89.5% when the model is trained on images with glasses.

If including multiple drivers during training helps to process images of those drivers, including images with and without glasses may help process images with and without glasses. We therefore trained models for each of the drivers using images with and without glasses. Figure 8b shows that a model trained on both types of images (with and without glasses) performs well on images with or images without glasses. Accuracy does not differ between a model trained on images with just glasses, tested on images with glasses, and a model trained on images with and without glasses ($\chi^{2}$(1) = 0.37, *p* = 0.54).

While training of the models requires a graphical processing unit (GPU), applying the model to images (’inference’) can be performed on a central processing unit (CPU). To obtain an estimate of the inference time per image, we ran one of the models over a total of 43,689 images on an i5-4570 CPU at 3.20 GHz. A minimum inference time of 0.0111 s, a maximum of 0.504 s, a median of 0.0124 s, and a mean of 0.0143 s were found. Using the median, this indicates that around 80 images per second can be processed, well above the typical frame rate of most cameras. These inference times are longer than most inference times reported by Vora et al. [49], but they ran their inference on a Titan X GPU, which most likely accelerated inference substantially. Note that for the present application (annotation of already collected driver images) real-time inference is not strictly required, but fast inference is always a nice feature when a large number of driver images need to be annotated.

### 4.2. Own Driver Dataset

**Figure 9:**
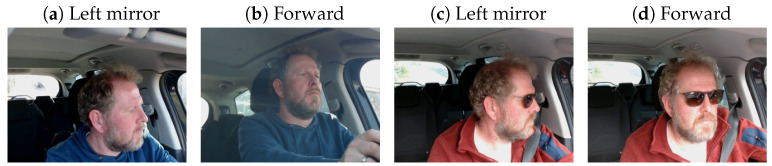
Examples of images of the male driver, with and without glasses, recorded with our own app.

**Figure 10:**
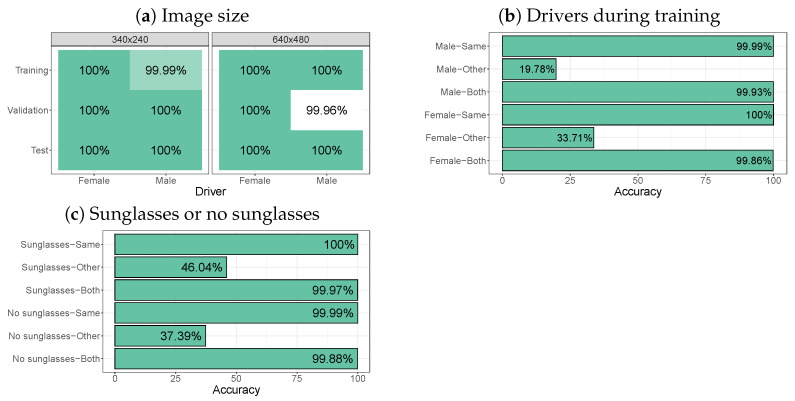
(**a**) Zone classification accuracy for the male and female driver for smaller (320 × 240) and larger (640 × 480) images (both without sunglasses). Each model was trained on that particular combination of driver and image size and then applied to the validation set (seen during training) and test set (not seen during training). (**b**) Accuracy per driver on a model trained with the same driver on a model trained with the other driver or a model trained on both drivers. Performance is computed across the training, validation, and test sets. (**c**) Accuracy for the male driver with or without sunglasses on a model trained with or without sunglasses or images with and without sunglasses (’Both’). Performance is computed across the training, validation, and test sets.

With our newly developed app, we collected images from two drivers (one male and one female) inside a stationary car using repeated gaze instructions to the drivers, as shown in Figure 9. Note that this dataset contains images from two viewing angles (recorded with two webcams) and it therefore examines whether the image classification method with YOLOv8 can deal with images from different viewing angles. Figure 10a shows that YOLOv8 provides a near-perfect zone classification performance for the twelve gaze zones in the dataset when the model was trained on the target driver. The left panel of Figure 10a shows that decreasing the image size to 320 by 240 pixels did not affect accuracy of the models. This means that reducing the image size can be an option if limited storage space or GPU capacity is available for training. Figure 10a also shows that the models perform well on yet unseen test images (which we did not test for the Lisa2 dataset).

In agreement with the findings for the Lisa2 dataset, Figure 10b shows that a model trained on the male driver does not perform well on the female driver, and vice versa. When a model is trained on both drivers (’both’) a near-perfect performance is restored for both the male and the female driver.

Also in line with the Lisa2 dataset results, Figure 10c shows that a model trained on the male driver with sunglasses does not perform well on images of the male driver without sunglasses, and vice versa. When the model is trained on the same type of images as it is applied to (’same’) or when the model is trained on both types of images, near perfect accuracy is restored. These results indicate that: (1) reducing the image size is a feasible strategy to deal with storage and GPU capacity issues and (2) the model should be trained on the types of images that need to be annotated.

### 4.3. Own Living Room Dataset

**Figure 11:**
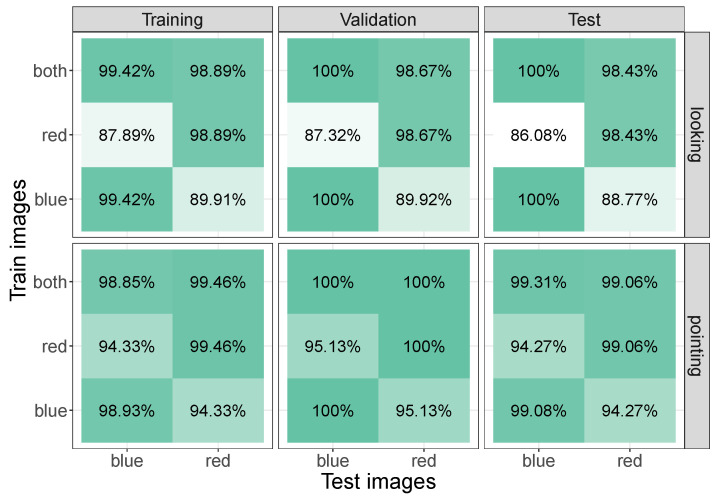
Zone classification accuracy for when an actor was looking or pointing at objects inside a living room. In between recordings, the actor changed from a red to a blue jacket, or vice versa. The change of the jacket reduced accuracy by around 5% (pointing) to 10% (looking) if these images were not included during training (’both’ refers to when both red and blue jacket training images were included).

We finally collected data inside the living room of one of the authors to examine whether the app and YOLOv8 classification can also be used in other contexts. Figure 11 provides a summary of the findings. The top row shows the classification accuracy for the looking images (the actor looking at one of ten different objects inside the room), whereas the bottom row shows the accuracy for the pointing images (the actor pointing at one of the same ten objects inside the room). In between recordings the actor changed the jacket that they were wearing from red to blue, or vice versa. Note that we only applied looking models to looking images and pointing models to pointing images (we were not examining whether we could classify looking behaviour with a model trained on pointing behaviour).

The data confirm previous observations with the other datasets. First, they show that the app and YOLOv8 can also be used for looking in other contexts and for classifying pointing movements. Second, changing the jacket leads to a small reduction in accuracy (in the order of 5% to 10%—worse for looking than for pointing), which can be restored by training on images with both types of jackets.

## 5. Apps

**Figure 12:**
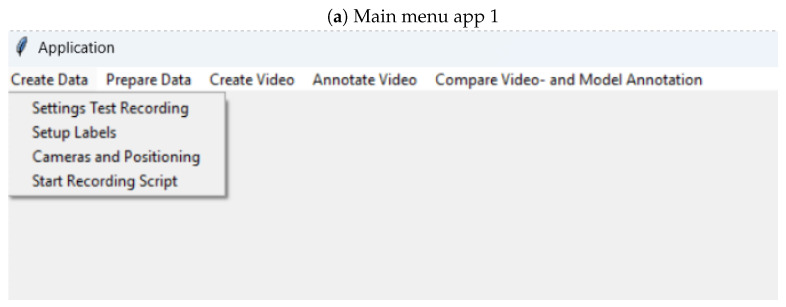

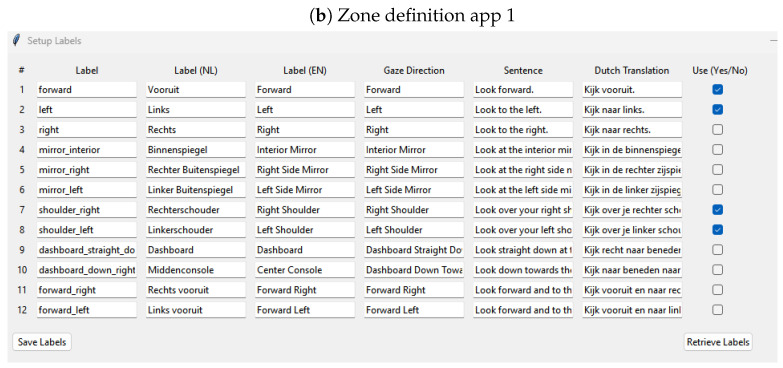
Screenshots from the first app that can be used to instruct participants to look at particular gaze zones and to collect images from the webcam, to extract frames, and structure the images into the folders for image classification. Note that a section of the window is shown in both images for better visibility.

We developed two apps to aid road safety researchers with the process of collecting the images (app 1 is available as a Python application from https://osf.io/6ahqd/?view_only=69ca1ce421f345e1809d77504efb8741, accessed on 10 November 2024), training an image classifier with YOLOv8 (app 2 is available as a packaged MacOs and Linux application from https://github.com/charmaine211/AutomaticDriverGazeDetection/releases/tag/v1.0.0, accessed on 10 November 2024), and applying the trained model to images or a video (both apps). The first app (Figure 12a) can be used to instruct drivers to look at a particular gaze zone (Figure 12b) and to record videos of the driver. This can be done in a stationary setting, or while driving. While driving, the driver is asked to name the target. Offline, the Whisper package [73] is used to tag the frames for that target (similar to Speak2label [43] but without the pre-processing, which we found worked well for spoken numbers). The app has options to select and test the webcams and to set up labels for the instructions and the number of repetitions for each the instructions. In a second step, video frames can be extracted from these videos and distributed across folders for training, validation, and test in the correct structure for YOLOv8.

**Figure 13:**
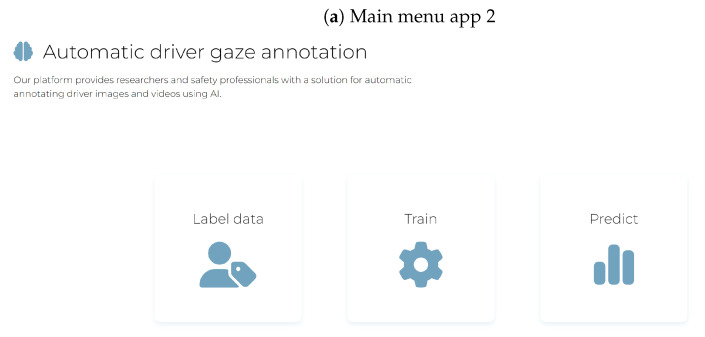

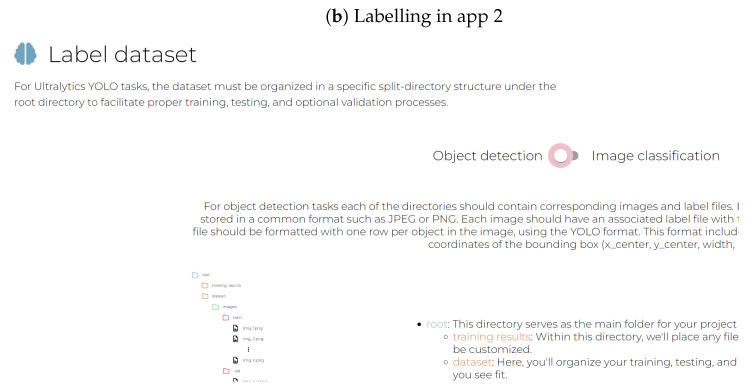
Screenshots from the second app that can be used to train the models and to generate the required file structure and annotations for object detection. Note that we did not use the object detection functionality in the present tests, because it is computationally more expensive and the image classification reached a near-perfect performance. Each image shows a section of the original screen for better visibility.

The second app (Figure 13a) has several functionalities. It can reformat the output (Figure 13b) from the first app into a format required for object classification (detecting the face for different gaze zones) by using DeepGaze [74]) and create the required configuration file for training with object detection. The second app also assists with the training process, saving the trained model, and applying the trained model to images and videos.

We evaluated the apps by asking naive users (three users for app 1 and four users for app 2) to perform a series of tasks with the apps while thinking aloud. For the first app, this showed that, in addition to a written manual, we also need to develop instruction videos for the apps. The user testing of the second app showed that users also need to be given the relevant background and terminology around computer vision tasks, such as the concepts of training, validation and test sets, models, training loss, and number of epochs. For both types of instructions, we will record instruction videos.

As demonstrated with our living room data, the apps can be used more broadly in other contexts. For example, the apps can also be used for gaze zone classification for different regions on a computer screen, or to determine the gaze zone for some someone standing in a space looking at different regions inside that space. The apps can also be used for recording images of someone’s hand and face for sign language recognition, and for collecting images and training a model for fatigue detection (instead of ’look left’ the instruction would then be something like ’yawn’ or ’look tired’) or other types of distraction (like using one’s phone or eating).

## 6. Discussion

The present study examined whether individual YOLOv8 image classification models provide an easy-to-use but accurate method to annotate the gaze zone where drivers look during naturalistic driving studies [3,20,75]. We tested the method on three (small) datasets. These included five drivers with and without glasses from the Lisa2 dataset [36], images of two drivers, one with and without sunglasses in a stationary car, and images of a person looking and pointing at targets inside a living room. For all three datasets, we found near-perfect gaze zone classification as long as the training data were aligned with the test data. For example, when a model was trained on drivers 1 and 2 from the Lisa2 dataset, it could correctly classify images from drivers 1 and 2 from that dataset, but not from driver 3, 4, or 5. Only when enough drivers were entered during training (at least four drivers), extrapolation to the remaining drivers took place.

Something similar was found when we used the images of the Lisa2 dataset with glasses [68]. When trained on driver 1 with glasses, gaze zone performance was near perfect for glasses, but substantially lower for driver 1 without glasses, and vice versa. When a model was trained on a driver with glasses and no glasses, classification performance was near perfect for both glasses and no glasses. This means that if you expect the driver to sometimes wear glasses and sometimes drive without glasses, you should have both types of images in your training dataset. In practice, this means that you have to ask your driver to look at gaze zones at least twice (with and without glasses). Note that we directly applied YOLOv8 image classification onto the images with glasses without any pre-processing. This contrasts with previous studies, where generative adversarial networks (GANs) were used to first remove the glasses from the images [36,68,70].

We achieved a higher accuracy for drivers with glasses than a previous study (that achieved around 80% correct [36]). This other study, however, focused on developing a single model for all drivers, whereas we focused on separate models for each driver, which is a less challenging task. Moreover, the past study used daytime and nighttime images, whereas the present study only used daytime images. Future work should therefore establish how well YOLOv8 performs on nighttime images. On images without glasses, it seems that YOLOv8 outperforms image classification with Resnet50 (around 91% [49]), Alexnet (around 89% [49]), and VGG16 (around 93% [49]), suggesting that YOLOV8 may be better suited for driver gaze zone detection than these previous networks.

Similar results were obtained for our own images, where a male driver was recorded with and without sunglasses. Sunglasses, in particular, make it impossible to detect the features of the eye (so eye gaze direction cannot be extracted). This means that the model must rely on the body posture and head direction to determine the gaze zone [76]. When images of the driver with sunglasses were included in the training set, the model could classify the gaze zone with sunglasses almost perfectly. Models that rely on features of the eyes would probably struggle under these conditions (and would need to fall back on the facial and body landmarks).

The near-perfect classification performance for models trained on images with glasses (Lisa2) and sunglasses (our own data) suggests that gaze shifts were performed in tandem with the eyes, head, and body. This seems to suggest that all the drivers in our datasets shifted their gaze mostly with their heads and less with their eyes (in terms of a previous paper, the drivers were more like owls and less like lizards, [17]). Studies of how people coordinate their head and eye movement during gaze shifts while driving [77] can be used to decide when to also focus on the eye region for better zone classification.

With our own data, we also demonstrated that a near-perfect classification performance can be maintained when smaller images (320 × 240 pixels) are used. This means that even with a lower-resolution camera, of after scaling down the images to reduce storage requirements, the model can still extract enough information from the smaller images for classification. Lower-resolution images also have the advantage that less GPU RAM is required to train the models. If GPU RAM is an issue, one could therefore try to reduce the images in size.

We found an excellent classification performance for eye region crops (Lisa2 dataset), head and upper body images (our own driver data and our looking data), and upper body images (pointing task). This shows that image classification with YOLOv8 can deal with large variations in the captured region. This is an advantage over methods that require the detection of certain eye region of facial features, which may struggle with images with occlusions of the eye region and reflections by glasses [18] (we did not check our images for blinks, so it is likely that these were included in the training, validation, and test sets).

A possible practical issue that we encountered with the images collected inside a living room was that a change of the jacket of the person affected the performance of the model (which was restored by training on images with both jackets). This means that if you expect your driver to change clothing, hairstyle, or accessories during or across driving sessions, it is important to include all those situations during training.

This leads to the possible issue of changing illumination during driving [18,36,49,50,51,63,76], as this is also expected to affect model performance. To combat this issue, the training set should include images under different illumination conditions. These could be obtained during driving, using a method such as Speak2Label [43], or by manual annotation of naturalistic driving images (but this has the risk of incorrect labelling [26]). Alternatively, data augmentation methods could be used to create different versions of the original images, for example, by changing the contrast, applying a gamma correction, or changing the brightness. Additionally, GAN methods [78] can be used for nighttime-style transfer onto daytime images. Alternatively, one could opt for (near) infra-red cameras [17,39,48,62,63,65,68], because the image classification method from YOLOv8 can also be used on such images and infra-red images tend to be less affected by the lighting conditions.

To address the question whether our method can deal with naturalistic driving conditions, it is important to first establish an accurate method to determine the gaze zone during naturalistic driving conditions. Human annotation is not sufficiently reliable [26] and it is unclear whether Speak2Label [43] results in similar gaze behaviour as found during unconstrained driving (and it may generate images of a driver who is speaking). Ideally, a remote eye tracker (so that images do not contain drivers with eye tracking glasses, speaking drivers, or drivers wearing a laser pointer on their head) should be used to obtain the ground truth for a dataset that is made available for benchmarking the various models [47].

When looking up the various existing datasets [12,38,43,44,52,53,54,55,56], we found that it was often difficult to obtain these datasets. We therefore also presented a simple tool (our app 1, built in tkinter in Python) that can be run on a laptop with one or more webcams attached. This tool can help researchers collect their own dataset. It can also help road safety researchers to perform the proposed model calibration for their drivers. Moreover, the tool can be used for collecting relevant data for driver activities other than looking in different gaze zones, such as using one’s phone to make a call, to write or read a text message, to eat or drink at the steering wheel, or to talk to a passenger.

One possible complication of the use of gaze zones (or other categories for different types of distraction) is that drivers may engage in gaze behaviour that does not fall into one of the zones (or engage in activities that were not recorded for training the model, such as talking to a passenger and using one’s phone). This may also be the case when drivers shift between one gaze zone to another. The YOLOv8 model [32] provides the top-5 probabilities that may be used to detect such out-of-zone activities. If the top-5 probabilities are similar (or when the top-1 probability is relatively low), this may present a situation where the driver is engaged in a behaviour that was not recorded for training the model. Future work should determine the optimal way of using these probabilities for this purpose.

We only examined how well YOLOv8 performed on the various datasets without making a comparison with other methods that, for example, used extra facial landmarks and head direction estimates. The reason for focusing on YOLOv8 was that we were aiming for a method that is easy to use (i.e., no pre-processing of the images and a few lines of code to train and apply a model), so that it would be feasible for a broad range of researchers. We showed that this simple approach can achieve high accuracy as long as the drivers and driving conditions overlap between training and test sets. Future research should establish how well YOLOv8 image classification works for the general case of multiple drivers and multiple conditions. As suggested by Camberg and colleagues [47], image classification methods, such as YOLOv8, may provide a good classification performance as long as sufficient images are available during training.

## 7. Conclusions

Road safety researchers have requirements for annotating gaze zones that are different from ADAS developers. We here show that near-perfect classification accuracy can be obtained by training a YOLOv8 image classification model specifically for a driver and the expected conditions (e.g., with or without glasses or sunglasses). We also provide two tools that road safety researchers can use to collect the required images and to train and apply the model. The current results only apply to recordings inside a stationary vehicle, and future research should examine how well they extend to naturalistic driving conditions.

## Figures and Tables

**Table 1:** Instruction sentences for the 12 targets inside the stationary car.

Zone	Instruction
1	Look forward
2	Look left
3	Look right
4	Look at the interior mirror
5	Look at the left side mirror
6	Look at the right side mirror
7	Look over your left shoulder
8	Look over your right shoulder
9	Look straight down at the dashboard
10	Look down to the centre console
11	Look forward and to the left
12	Look forward and to the right

**Table 2:** Ten targets around the computer screen to which the webcam was attached for the living room data collection.

Zone	Description
1	Webcam on top of the screen
2	Phone left of the screen on the table
3	Notebook right of the screen on the table
4	Computer left of the screen on the same table
5	Door handle right of the screen
6	Door stop on the floor right of the screen
7	Plant far left of the screen
8	Picture above the screen
9	Socket left and above the screen
10	Cup in front on the screen on the table

## Data Availability

Images, trained models, analysis code, and the first app can be downloaded from: https://osf.io/6ahqd/?view_only=69ca1ce421f345e1809d77504efb8741, accessed on 10 November 2024.
